# The Relationship between Multiple Health Behaviours and Brachial Artery Reactivity

**DOI:** 10.1155/2012/846819

**Published:** 2012-02-29

**Authors:** Jennifer L. Gordon, Kim L. Lavoie, André Arsenault, Bernard Meloche, Blaine Ditto, Tavis S. Campbell, Simon L. Bacon

**Affiliations:** ^1^Montreal Behavioural Medicine Centre, Montréal, Québec, Canada; ^2^Research Centre, Montreal Heart Institute, A University of Montreal Teaching Hospital, Montréal, Québec, Canada H1T 1C8; ^3^Department of Psychology, McGill University, Montréal, Québec, Canada H3B 1B1; ^4^Research Centre, Sacré-Cœur Hospital of Montreal, A University of Montreal Teaching Hospital, Montréal, Québec, Canada H4J 1C5; ^5^Department of Psychology, University of Québec at Montréal (UQAM), Montréal, Québec, Canada H3C 3P8; ^6^Department of Psychology, University of Calgary, Calgary, Alberta, Canada T2N 1N4; ^7^Department of Exercise Science, Concordia University, 7141 Sherbrooke Street West, SP165.35, Montréal, Québec, Canada H4B 1R6

## Abstract

*Background*. The effects of smoking, alcohol consumption, obesity, and a sedentary lifestyle on endothelial function (EF) have only been examined separately. The relative contributions of these behaviours on EF have therefore not been compared. *Purpose*. To compare the relative associations between these four risk factors and brachial artery reactivity in the same sample. *Methods*. 328 patients referred for single-photon emission computed tomography (SPECT) exercise stress tests completed a nuclear-medicine-based forearm hyperaemic reactivity test. Self-reported exercise behaviour, smoking habits, and alcohol consumption were collected and waist circumference was measured. *Results*. Adjusting for relevant covariates, logistic regression analyses revealed that waist circumference, abstinence from alcohol, and past smoking significantly predicted poor brachial artery reactivity while physical activity did not. Only waist circumference predicted continuous variations in EF. *Conclusions*. Central adiposity, alcohol consumption, and smoking habits but not physical activity are each independent predictors of poor brachial artery reactivity in patients with or at high risk for cardiovascular disease.

## 1. Introduction

Poor endothelial function (EF) predicts future cardiovascular events [1] and is thought to be influenced by several behavioural risk factors for cardiovascular disease (CVD) including smoking, physical activity, obesity, and alcohol consumption. Thus, poor EF may mediate some of the risks for CVD created by these behaviours. For example, current smokers have been found to have lower EF than those who have never smoked [2–6]. Furthermore, smoking has been shown to induce an acute decrease in EF [4, 7]. To our knowledge, however, there has only been one study that has examined the relationship between lifetime smoking and EF [8]. While this study found that pack/years were inversely related to EF, the generalizability of these results may be limited given the narrow age range of its participants (20–28 years). 

Cross-sectional studies have found that sedentary participants display reduced EF compared with athletes [9–11], though inconsistencies have been noted [12, 13]. While some interventions to increase physical activity have been found to have a positive impact on EF [12, 14–24], others have failed to do so [25–28]. To our knowledge, only two studies have examined the relationship between EF and physical activity in moderately active participants; one study found a significant effect of physical activity on EF in children [29], while the other did not find a relationship in adults [30].

While obesity is influenced by several factors, including physical activity, caloric consumption has been found to be the most important predictor of weight loss or gain and caloric restriction to be the most effective means of inducing weight loss [31–33]. As such, although “obesity” is not a behaviour per say, here it will be considered a proxy of long-term excess caloric consumption. Cross-sectional studies have observed obese and overweight participants, classified according to BMI, have worse EF compared to normal weight controls [34–37]. Intervention studies using either gastric bypass surgery [38] or a low-calorie diet [39, 40] have also reported weight loss to result in improvements in EF. However, only one study to date has examined the relationship between obesity and EF in participants with a wide range of BMIs, finding that both overweight and obese individuals have worse EF than normal weight controls [41].

The effect of alcohol consumption on EF is equivocal. While studies comparing alcoholics to nonalcoholic controls have reported alcoholics to have significantly lower EF [42–44], the impact of moderate amounts of alcohol on EF is uncertain. Acute wine consumption has generally been observed to have a positive impact on EF [45–47] though one study found no difference in EF when pairing a glass of red wine versus a control beverage with a high-fat meal [48]. Furthermore, the equivalent of one drink's worth of ethanol has resulted in no acute change in EF [49], while four drinks' worth of ethanol resulted in a significant acute decrease in EF [50]. There has been only one published report on the relationship between moderate alcohol consumption and EF. In this study of 108 men with coronary artery disease, those who drank 1–3 drinks/day had significantly better EF than abstainers, suggesting an inverted J-shaped relationship between alcohol consumption and EF [51].

While the effects of smoking, physical activity, obesity, and alcohol consumption on EF have been examined, some important gaps in knowledge remain. First, there is a lack of research examining the relationship between lifetime smoking and EF. Second, further research is needed to clarify the nature of the relationship between moderate levels of physical activity and EF. Third, studies are needed to clarify the relationship between regular moderate alcohol consumption and EF. Fourth, since most studies on health behaviours and EF have been conducted in generally healthy noncardiac samples, this relationship should be explored in samples that have or are at risk for cardiovascular disease. Finally, since these variables often cooccur, research is needed to determine their independent effects. The primary goal of the current study was to examine the relationship between all four health behaviours and brachial artery reactivity, used as a proxy of EF, in a single sample. This allowed determination of whether each health behaviour had an impact on EF independent of the others and allowed evaluation of their relative impact on EF (Table 1).

## 2. Methods

### 2.1. Participants

The study was a substudy of the Mechanisms and Outcomes of Silent Myocardial Ischemia (MOSMI) study, a longitudinal study of risk factors for silent ischemia and the impact of silent ischemia on cardiovascular outcome. A total of 904 consecutive patients referred for single-photon emission computed tomography (SPECT) exercise stress tests between July 2005 and December 2006 in the Nuclear Medicine Service of the Montreal Heart Institute were recruited. To be eligible for the MOSMI study, patients had to be undergoing SPECT exercise stress testing, be over 18 years old, and speak either English or French. Patients were excluded if they had experienced a cardiac event (e.g., myocardial infarction) in the last 4 weeks, if they had a more prominent medical condition than CVD (e.g., cancer, chronic obstructive pulmonary disease), or were pregnant or nursing. A subsample of 328 patients from the MOSMI study underwent EF testing. The selection of this group from the main cohort was done based on the random allocation to available testing time slots. There were no differences in age, sex, or CVD status between this subsample and those patients recruited for the MOSMI study but who were not included in final analysis. The MOSMI study was approved by the Human Ethics Committee of the Montreal Heart Institute and written informed consent was obtained from all participants.

### 2.2. Procedure

Patients presenting to the Nuclear Medicine Service of the Montreal Heart Institute on the day of their exercise stress test were approached to participate in the MOSMI study. Once consent was obtained, demographic and medical information, including health behaviours, was collected using a self-report questionnaire and waist circumference was measured. Patients then underwent standard treadmill exercise stress testing (Bruce protocol) followed by SPECT imaging. As per the SPECT protocol, all patients returned the following day to complete their rest scan. Prior to the rest scan patients underwent the forearm hyperaemic reactivity (FHR) test to measure brachial artery reactivity in response to hyperaemic challenge. All SPECT imaging was conducted according to standard procedure [52–54]. In general, patients were maintained on their usual medication, the only exception being that people were asked to withhold taking beta-blockers on the day of the exercise and rest SPECT studies.

### 2.3. Brachial Artery Reactivity Assessment

The FHR technique, implemented by a trained Nuclear Medicine technician, was used to assess brachial artery activity. FHR is considered a marker of EF [55, 56]. FHR measures alterations in the brachial artery endothelium following a hyperaemic challenge [57, 58]. The patient is seated with both arms extended over the top of a standard large field-of-view gamma camera with a low negative high resolution collimater (Scintronix, London, UK) facing upward, hands prone. To create the hyperaemic challenge, a blood pressure cuff (Adult First Responders, B&A Instruments, New York, New York) was placed on the right arm and inflated at 50 mmHg above systolic blood pressure for 5 minutes, after which it was released. Forty-five seconds after the cuff was deflated, 0.42 mCi/kg of the radioactive tracer Tc-99m-tetrofosmin (Myoview, Amersham Health, Princeton, NJ) was injected into the patient's arm via a small catheter positioned in the bend of the left arm. For the next 10 minutes, dynamic image acquisitions were realized using 128 × 128 matrices at a sampling rate one frame per second. From this, the ratio between the rate of blood flow into the right (the hyperaemic arm) and left (the control arm) arms, the relative uptake ratio (RUR), was calculated using custom-made software (Sygesa, Montreal, Canada). The higher one's RUR, the better the endothelial tissue is at responding to the hyperaemia, via the promotion of vasodilation. Thus, a higher RUR indicates better EF and a lower RUR indicates worse EF. More specifically, an RUR under 3.55 is considered to be indicative of endothelial dysfunction [58]. This technique has a high level of reproducibility with excellent intra- and interrater reliability [59].

### 2.4. Health Behaviours

#### 2.4.1. Physical Activity

Leisure time physical activity was derived from a slightly adapted 12-month version of the Physical Activity Recall Interview [60]. Specifically, given the nature of activity patterns in Montreal, separate details were collected for winter-like and summer-like activities and then combined. Physical activity levels were calculated as average metabolic equivalents MET-hrs/week.

#### 2.4.2. Smoking

Lifetime cigarette consumption was assessed using the standard calculation of pack/years (average number of packs smoked/day × number of years having smoked) [61]. Patients were also classified as current, ex-, and never smokers.

#### 2.4.3. Obesity

As centrally distributed fat seems to be more significant in the aetiology of CVD [62], waist circumference was used as our measure of central adiposity. Prior to the EF test, the attending technician measured the participants' waist circumference at the mid-point between the super-illiac crest and the lower thoracic cavity.

#### 2.4.4. Alcohol

Participants were asked to report their usual daily or weekly consumption of alcoholic beverages. Using this data, weekly units of alcohol consumed were estimated.

### 2.5. Data Analysis

Our missing data analysis procedures used missing at random (MAR) assumptions, as per Rubin's rules [63]. We used the PROC MI method of multiple multivariate imputation in SAS. We independently analyzed 5 copies of the data, each with missing values imputed, and then used PROC MIANALYZE to average estimates of the variables to give a single mean estimate and adjusted standard errors according to Harrell's guidelines [64]. Details of the amount of missing data per variable are included in Table 2. 

To examine the contributions of physical activity, smoking, obesity, and alcohol consumption to EF, a series of GLMs on the individual variables was also conducted, first for the individual health behaviours alone, second with the individual health behaviours and covariates, and third for all four behaviours together and the covariates. Since a nonlinear relationship was expected to exist between alcohol consumption and EF, participants were split into one of three groups in terms of their alcohol consumption. Group 1 consisted of heavy drinkers (defined as 22 or more drinks per week), group 2 consisted of abstainers, and group 3 consisted of moderate drinkers (1–21 drinks per week). The groups were defined as such since consuming 1–3 drinks per day has been associated with better EF than abstinence [51], while 4 or more drinks have been found to negatively impact EF acutely [50]. It was therefore expected that heavy drinkers would have the worst EF and moderate drinkers would have the best EF, creating a linear relationship between group number and EF. Age, sex, CVD status (CVD was defined as having a previous myocardial infarction, stroke, angioplasty, or coronary artery bypass graft), ace inhibitor use, statin use, hypertension, diabetes, and hypercholesterolemia were chosen as covariates *a priori*, as all have been shown to have strong independent effects on EF [7, 65–71]. To examine potential interactions between the health behaviours an interaction term for each pair of continuous health behaviours was included one at a time in a GLM including the above-mentioned covariates.

To examine possible associations between the health behaviours and risk of having endothelial dysfunction, defined as an RUR under 3.55, two logistic regression analyses were conducted predicting endothelial dysfunction from all four health behaviours and the covariates together. For these analyses, waist circumference was split into tertiles: <92 cm, 92–108 cm, and >108 cm. Smoking was defined by smoking status (current/past/never). Alcohol consumption was split by the groups described above. Finally, physical activity was split into tertiles: 0, 0–9.7 and >9.7 MET-hrs/week.

## 3. Results

The characteristics of the 328 participants are presented in Table 2. The GLM analyses for individual health behaviours revealed that waist circumference (*β* (SEM) = −.03 (.01), *P* < .0001) contributed significantly to EF. A trend for an effect of smoking on EF measured using pack-years (*F* = 1.78, *P* = .075) was also found. However, physical activity (*β* (SEM) = −.01 (.01), *P* = .528), smoking status (*β* (SEM) = −.22 (.15), *P* = .131), and alcohol consumption (*β* (SEM) = .19 (.12), *P* = .136) were not associated with EF as a continuous variable.

GLMs for the individual health behaviours with age, sex, CVD status, ace inhibitor use, statin use, hypertension, diabetes, and hypercholesterolemia as covariates were then conducted. These analyses revealed that waist circumference (*β* (SEM) = −.04 (.01), *P* < .0001) contributed significantly to the variability in EF, while physical activity (*β* (SEM) = −.01 (.01), *P* = .398), alcohol consumption (*β* (SEM) = −.17 (.13), *P* = .167), and smoking measured according to pack-years (*β* (SEM) = −.01 (.01), *P* = .203) or smoking status (*β* (SEM) = −.21 (.15), *P* = .153) did not.

**Figure 1:**
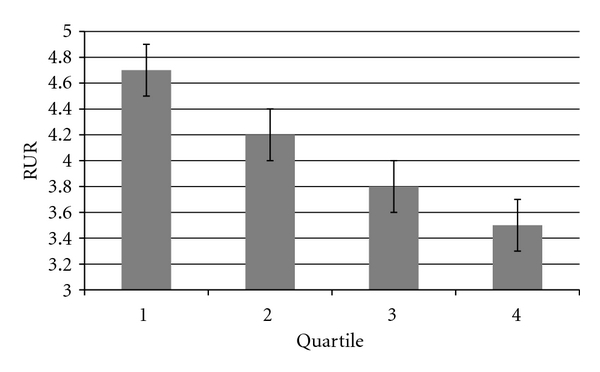
Mean relative uptake ratio (RUR) as a function of waist circumference split into quartiles, with standard error bars, adjusting for covariates, smoking, physical activity, and alcohol consumption.

Results of a GLM with all covariates and health behaviours included revealed that waist circumference was a significant independent predictor of EF considered as a continuous variable (Table 3 and Figure 1). Neither alcohol consumption, physical activity, nor smoking, measured according to pack years were significantly related to EF (Table 3). Comparable results were found when lifetime smoking was replaced by current smoking status.

The results of the GLMs separately examining the interaction between each pair of health behaviours revealed no significant interaction effects.

**Figure 2:**
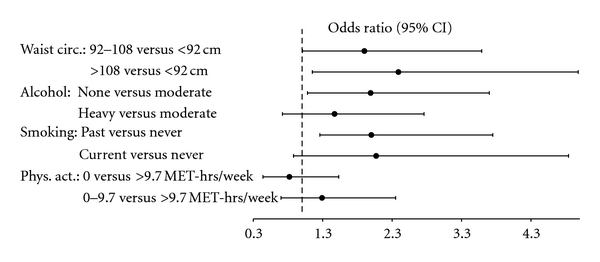
Forest plot of odds-ratios and 95% confidence intervals for the effect of each health behaviour on endothelial dysfunction adjusting for covariates and the other health behaviours.

A logistic regression analysis, including all covariates and health behaviours in the model revealed that in comparison to the lowest tertile of waist circumference (<92 cm), being in the second tertile (92–108 cm) was associated with a 90% increased risk of endothelial dysfunction (OR = 1.90, 95% CI = 1.01–3.59). Being in the third tertile for waist circumference (>108 cm), on the other hand, was associated with more than double the risk of endothelial dysfunction (OR = 2.39, 95% CI = 1.15–4.98). Alcohol consumption group was also a significant predictor of endothelial dysfunction such that being a nondrinker versus a moderate drinker was associated with nearly double the risk of endothelial dysfunction (OR = 1.99, 95% CI = 1.08–3.70), while the risk associated with being a heavy drinker did not differ from being a moderate drinker (OR = 1.57, 95% CI = 0.82–2.86). Being a past smoker versus a never smoker was associated with double the risk of endothelial dysfunction (OR = 2.00, 95% CI = 1.26–3.75), while the risk associated with being a current smoker did not reach significance (OR = 2.17, 95% CI = 0.88–4.84). Finally, the odds ratios for being in the first (OR = 0.82, 95% CI = 0.44–1.53) or second tertile (OR = 1.29, 95% CI = 0.70–2.35) in comparison with the third tertile for physical activity were not significant (Figure 2).

## 4. Discussion

This study is the first attempt to assess the effects of waist circumference, physical activity, alcohol consumption, and smoking on EF in a single sample of cardiac patients. The GLM analyses revealed that waist circumference was negatively associated with continuous variations in EF and that there was a trend for a significant negative association between lifetime smoking and EF when they were each included in the model alone. However, when covariates were added to the model, this trend disappeared but the significant effect of waist circumference on EF remained. When all four health behaviours were included in the same model with the covariates, only waist circumference was significantly associated with EF considered as a continuous variable. Additional logistic regression analyses, including all four health behaviours and covariates together, revealed that increased waist circumference also increased risk for clinical endothelial dysfunction. Surprisingly, abstinence from alcohol and past smoking predicted endothelial dysfunction, while heavy alcohol consumption and current smoking did not.

 The current findings suggest that central adiposity is negatively associated with EF. Consistent with the current findings, several studies have found measures of central adiposity such as waist circumference and waist-hip ratio, to be predictors of cardiovascular outcomes in patients both with [72] and without [73–77] known CVD. Although the exact mechanism behind this obesity-induced increase in endothelial dysfunction is unclear, two candidate processes have been proposed: (1) via alternations in adipokine levels; and (2) via increases in free fatty acid levels. In brief, it would appear that increasing levels of obesity are associated with increases in adipokines that negatively influence the endothelium [78], and decreases in adipokines that exert positive effects on the endothelium [79]. For example, leptin, which is increased in obese patients, has been shown to increase the release of reactive oxygen species in human endothelial cells [78], thus diminishing the bioavailability of nitric oxide (NO), an important vasodilator and inhibitor of inflammation and platelet aggregation. Alternatively, adiponectin, which is downregulated in obese individuals [79], stimulates nitric oxide production, and promotes vasodilation [80]. Recent studies have also found increased levels of endothelial dysfunction when free fatty acids, which are abundant in patients with abdominal obesity [81], were administered exogenously [82, 83]. This alteration is thought to occur by free fatty acids decreasing endothelial nitric oxide synthase (eNOS) activity [82].

The findings regarding cigarette smoking and alcohol use are somewhat more complex. That is, GLM analyses indicate that these variables were not associated with continuous fluctuations of EF. However, the logistic regression analyses reveal that past (but not current) smoking and abstinence from alcohol (but not heavy consumption) both increased the risk of endothelial dysfunction. The fact that the odds ratios for current smoking did not reach significance may be partially due to small sample sizes since relatively few participants were current smokers. The finding that past smoking was associated with an increased risk of endothelial dysfunction suggests that smoking-induced endothelial damage is long lasting. This is consistent with previous research finding that the somewhat improved flow-mediated dilation of former smokers was not statistically greater than current smokers [6]. Smoking is thought to damage endothelial cells directly [84] and to increase oxidative stress, thereby decreasing nitric oxide availability [85]. Smoking also appears to reduce the immune system's ability to repair this endothelial damage by decreasing the number of circulating endothelial progenitor cells, which are mobilised in response to vascular injury [86]. Following smoking cessation, the number of circulating endothelial progenitor cells has been found to increase rapidly [87], suggesting that the EF of ex-smokers should begin improving soon after quitting. However, it is unknown to what extent the length of time of smoking or the length of time since quitting impacts current EF. It may therefore be the case that the ex-smokers in the current study had either smoked too long or had not been smoke-free long enough to exhibit fully recovered EF.

Our finding that heavy alcohol consumption is not associated with greater risk of endothelial dysfunction is inconsistent with previous research finding that heavy consumption has been found to induce endothelial cell apoptosis by increasing oxidative stress [88] and impairing nitric oxide production [89]. This finding may therefore be related to sample size, considering the relatively small number of heavy drinkers in the current study. However, that moderate alcohol consumption is associated with better EF than abstinence is consistent with previous findings [51]. It is believed that moderate alcohol consumption may improve EF by increasing NO levels [90] and decreasing homocysteine levels, which is an amino acid causing oxidative stress [91].

That physical activity was not found to be associated with EF is inconsistent with studies comparing sedentary individuals with athletes [9–11] but is consistent with the one study that has examined the relationship between moderate physical activity and EF in adults [30]. This finding suggests that physical activity may contribute little to EF among moderately active individuals at high risk for cardiovascular disease who likely engage in several poor health behaviours at once. Considering the average participant in this study does not meet the minimum requirements for physical activity set by the American College of Sports Medicine and the American Heart Association [92], which is between 7.5 and 12.5 MET-hrs/week, greater levels of physical activity may be needed to see its effect on EF.

 Of course, the results of the current study must be considered in light of some limitations. As with all cross-sectional studies, it is impossible to draw conclusions about the cause and effect relationship between the health behaviours assessed and EF. The current study also used retrospective self-report measures for physical activity, alcohol consumption, and smoking, which may be susceptible to recall bias. Drink type was also not taken into account for alcohol consumption, which is problematic given that wine has been found to have a unique beneficial effect on EF [47, 93]. Our failure to record patients' use of nicotine replacement therapy, known to affect EF [94], is also a limitation.

While the current study has its limitations, the fact that it examined the relationship between EF and smoking, obesity, physical activity, and alcohol consumption in the same statistical model is an important and unique strength. While other studies that have examined the effect of only one health behaviour on EF risk overestimating the portion of variance in EF explained by the behaviour, the present study should more accurately estimate the independent effect of each health behaviour on EF. This is particularly true since poor health behaviours tend to aggregate. It is also a strength that this study examined the effect of health behaviours on EF in a sample of patients at risk for cardiovascular disease since poor health behaviours would be expected to have the most important and imminent impact in this population. Other strengths of this study include its large sample size, the inclusion of several important covariates, and the quality of the health behaviour measures used. Finally, despite the relative novelty of the FHR technique, it has been shown to be highly reliable [59] and reproducible [58, 95].

 In conclusion, the results of this study indicate that central adiposity and smoking are independently associated with worsening EF, while moderate alcohol consumption appears to have an independent positive effect on EF. Physical activity, on the other hand, does not seem to be associated with EF when controlling for other health behaviours and relevant covariates. These findings may imply that within a population exhibiting numerous risk factors for cardiovascular disease, weight reduction, smoking cessation, and moderate alcohol consumption should be emphasized and prioritized over increasing physical activity as interventions to improve patients' cardiovascular profiles. This prioritization may be particularly helpful given the difficulty with which individuals change just one health behaviour. Further work is needed to confirm the mechanisms by which central adiposity, smoking and alcohol consumption influence EF. Further research is also needed to examine the effect of health behaviours on EF over a longer period of time to examine how EF changes as a function of health behaviours over time. Future research should also aim to assess the extent to which the relationship between health behaviours and cardiovascular disease is explained by the impact of health behaviours on EF. 

## Figures and Tables

**Table 1:** Summary table.

What is known about the topic	What this study adds
(i) Poor endothelial function is an early predictor of future cardiovascular events. (ii) Poor health behaviours, including smoking, sedentary behaviour, obesity, and excessive alcohol consumption, have individually been associated with endothelial dysfunction. (iii) These studies have generally compared “extreme” behaviours (e.g., marathon runners versus sedentary individuals).	(i) There are independent effects of current smoking habits, obesity and alcohol consumption on brachial artery reactivity. (ii) Whilst any improvement in a single health behaviour might have a positive influence on endothelial function, targeting multiple behaviours should yield greater benefits.

**Table 2:** Participant characteristics.

Variable	*M* ± SD or % (*n*)	Data missing (%)
Age (yrs)	59.7 ± 9.6	1%
Sex (% women)	25% (83)	0%
Endothelial dysfunction (RUR < 3.55)	44% (145)	0%
Average # drinks/week	13.4 ± 12.8	8%
Waist circumference (cm)	99.6 ± 12.0	3%
Leisure-time physical activity (MET-hrs/week)	7.2 ± 12.4	1%
Lifetime smoking (pack years)	15.7 ± 19.6	9%
Smoking status	Current: 11% (37) Previous: 57% (188) Never: 31% (102)	0%
Statin use	49% (157)	3%
Ace inhibitor use	26% (82)	3%
Cardiovascular disease	41% (133)	0%
Diabetes	16% (38)	0%
Hypercholesterolemia	62% (198)	2%
Hypertension	60% (196)	0%

**Table 3:** Summary of multiple regression analysis for all health behaviours predicting brachial artery reactivity, controlling for age, sex, CVD status, ace inhibitor use, statin use, hypertension, diabetes, and hypercholesterolemia.

Variable	*β*	SE	*t*	*P*
Waist circumference	−0.03	0.01	−3.95	<.000
Physical activity	−0.01	0.01	−1.01	.313
Lifetime smoking	−0.00	0.01	−0.86	.392
Alcohol usage	0.14	0.12	−1.13	.258
